# Immunotherapeutic Value of MAP1LC3C and Its Candidate FDA-Approved Drugs Identified by Pan-Cancer Analysis, Virtual Screening and Sensitivity Analysis

**DOI:** 10.3389/fphar.2022.863856

**Published:** 2022-03-02

**Authors:** Xudong Zhang, Kunhang Li, Shiyu Zhong, Shengyu Liu, Tao Liu, Lishuai Li, Shuo Han, Qingqing Zhai, Nan Bao, Xin Shi, Yijun Bao

**Affiliations:** ^1^ Department of Neurosurgery, The Fourth Hospital of China Medical University, Shenyang, China; ^2^ School of Management, Shanghai University, Shanghai, China; ^3^ College of Medicine and Biological Information Engineering, Northeastern University, Shenyang, China; ^4^ School of Maths and Information Science, Shangdong Technology and Business University, Yantai, China; ^5^ Business School, All Saints Campus, Manchester Metropolitan University, Manchester, United Kingdom

**Keywords:** pan-cancer analysis, prognosis, immunotherapy, tumor microenvironment, molecualr docking

## Abstract

**Background:** The autophagy pathway within the tumour microenvironment can be regulated to inhibit or promote tumour development. In the fight against tumour growth, immunotherapy induces an anti-tumour immune response, whereas autophagy modulates this immune response. A key protein in the autophagy pathway, microtubule-associated protein 1 light chain 3 (MAP1LC3), has recently become a hotspot for tumour research. As a relatively novel member, the function of MAP1LC3C in tumours still need to be investigated. Therefore, the goal of this study was to look into the possible link between MAP1LC3C and immunotherapy for 33 kinds of human malignancies by using pan-cancer analysis.

**Methods:** High-throughput sequencing data from The Cancer Genome Atlas, Genotype-Tissue Expression Project and Cancer Cell Line Encyclopedia databases, combined with clinical data, were used to analyze the expression of MAP1LC3C in 33 types of cancer, as well as patient prognosis and neoplasm staging. Activity scores were calculated using ssGSEA to assess the MAP1LC3C activity in pan-cancer. Associations between MAP1LC3C and the tumour microenvironment, including immune cell infiltration and immunomodulators, were analyzed. Moreover, tumour tissue ImmuneScores and StromalScores were analyzed using the ESTIMATE algorithm. Additionally, associations between MAP1LC3C and tumour mutational burden/microsatellite instability, were investigated. Finally, based on the expression and structure of MAP1LC3C, the United States Food and Drug Administration (FDA)-approved drugs, were screened by virtual screening, molecular docking and NCI-60 drug sensitivity analysis.

**Results:** Our study found that MAP1LC3C was differentially expressed in tumour and normal tissues in 23 of 33 human cancer types, among which MAP1LC3C had prognostic effects in 12 cancer types, and MAP1LC3C expression was significantly correlated with tumour stage in four cancer types. In addition, MAP1LC3C activity in 14 cancer types was consistent with changes in transcription levels. Moreover, MAP1LC3C strongly correlated with immune infiltration, immune modulators and immune markers. Finally, a number of FDA-approved drugs were identified via virtual screening and drug sensitivity analysis.

**Conclusion:** Our study investigated the prognostic and immunotherapeutic value of MAP1LC3C in 33 types of cancer, and several FDA-approved drugs were identified to be highly related to MAP1LC3C and can be potential cancer therapeutic candidates.

## Introduction

Autophagy is a process that occurs in all eukaryotes, involving in the capture by an autophagosome and transport to the lysosomes for decomposition and recycling (Ohsumi, 2012; Morishita and Mizushima, 2019). Autophagy has long been considered as a non-selective process that meets the needs of cell synthesis and metabolism; however, recent studies have proved the existence of selective autophagy pathways, specifically targeting damaged organelles, pathogens and unfolded proteins (Le Guerroué et al., 2017). To better cope with stresses in the tumour microenvironment, the autophagy pathways in various cell types can be regulated to inhibit or promote tumour development (Xia et al., 2021).

The immune system plays an important role in preventing tumour occurrence, development and metastasis and in tumour treatment. Immune surveillance is a function of the immune system that helps to recognise, kill and remove tumour cells; however, tumour cells can evade tumour immunity through immunosuppressive responses (Zou, 2005). Moreover, immunotherapy fights against tumours by arousing the anti-tumour immune response in the immune system (Zou et al., 2016). Recent studies have shown that autophagy is involved in the various biological processes of immune cells (Clarke and Simon, 2019) and can modulate tumour growth by regulating the immune response (Yang et al., 2018).

A common key factor in both selective and non-selective autophagy pathways is the ubiquitin-like protein binding system, including the ATG5-ATG12 binding system and ATG8-lipidation system (Tooze and Yoshimori, 2010). Human cells contain at least six ATG8 family members, which can be divided into two subfamilies: MAP1LC3A (LC3A), LC3B, LC3C and GABARAP, GABARAPL1, GABARAPL2 (Slobodkin and Elazar, 2013). Microtubule-associated protein 1 light chain 3 (MAP1LC3), a ubiquitinated protein of the ATG8-lipidation system, is involved in various pathophysiological processes of the body. The role and mechanism of MAP1LC3 subfamily in a tumour is complex, making it a hotspot for tumour research. However, research on MAP1LC3C, a relatively new member, is still lacking. There are few articles referring to MAP1LC3C and no articles that deeply investigate the function of this gene in tumours. Therefore, a pan-cancer study of MAP1LC3C is necessary.

This study is the first to analyse the prognostic and immunotherapeutic value of MAP1LC3C in various cancer types. The expression and activity of MAP1LC3C and its correlation with patient prognosis and tumour stage in 33 human cancer types, combined with clinical information, were analysed using online databases such as TCGA, GTEx and CCLE. The effects of MAP1LC3C on the immune microenvironment, tumour mutational burden (TMB), microsatellite instability (MSI) and two immunotherapy biomarkers were studied using CIBERSORT and ESTIMATE algorithms. Finally, based on the expression and structure of MAP1LC3C, virtual screening and drug sensitivity analysis of the FDA-approved drug library were conducted to identify therapeutic drugs. Therefore, this study aims to elucidate the role of MAP1LC3C in the immune system and its impact on cancer prognosis and immunotherapy, as well as to provide some references for therapeutic agents.

## Methods

### Data Collection

Transcriptome expression profiles, clinical data and mutation data of 33 cancer types in the TCGA database were downloaded through Xena (Goldman et al., 2020). Control gene data of six cancer types (ACC, LGG, LAML, OV, TGCT and UCS) were obtained from the GTEx database (Consortium, 2020). Sequencing data of cell lines were obtained from the CCLE database (Nusinow et al., 2020). As this study used open data, ethical approval was not required.

### Identification of MAP1LC3C Differential Expression and Its Association With Clinical Characteristics

MAP1LC3C expression data were extracted from the TCGA and GTEx databases, and the gene expression levels between 33 cancer and normal samples were compared using R-package LIMMA (Ritchie et al., 2015). Additionally, the correlation between MAP1LC3C expression and tumour stage was explored. Combined with clinical information, the relationship between gene expression level and patient survival was calculated using univariate Cox regression. When the hazard ratio (HR) was greater than 1 (HR > 1), MAP1LC3C expression was considered as a risk factor. Kaplan–Meier (KM) analysis was performed to compare the overall survival (OS), disease-specific survival (DSS), disease-free interval (DFI) and progression-free interval (PFI) of patients with cancer, which was stratified by median MAP1LC3C expression. *p* < 0.05 was considered statistically significant for the analyses.

### Study of MAP1LC3C Activity

To further study the activity of MAP1LC3C in pan-cancer, genes that are closely related to MAP1LC3C as relevant genes of MAP1LC3C through the co-expression method were found, and the MAP1LC3C activity score of each sample was obtained using R-package GSVA and ssGSEA method (Hänzelmann et al., 2013). The differences in MAP1LC3C activity between the normal and tumour groups were studied, and the scores of MAP1LC3C activity in 33 cancer types were obtained.

### Correlation Analysis of MAP1LC3C Expression and Immune-Related Factors

The CIBERSORT algorithm was used to calculate the immune cell invasion level of each tumour sample (Newman et al., 2019). A total of 100 permutations were run using the LM22 signature. *p* < 0.01 and |R| > 0.4 indicated significant correlation. Immunoscores and stromal scores were calculated for each sample using the ESTIMATE algorithm (Yoshihara et al., 2013). Additionally, the list of immune modulators (Supplementary Table S1), including immune inhibitors, stimulators and major histocompatibility complex (MHC) molecules, were downloaded from the TISIDB database (Ru et al., 2019).

## Virtual Screening and Molecular Docking

Structural information on 2858 FDA-approved and pharmacopeial drugs was downloaded from the TargetMol (Inc.A-Approved &, 2021). The spatial structure information of the MAP1LC3C protein (PDB 2NCN) was downloaded from the Protein Data Bank (PDB) database (Berman et al., 2000). The GHECOM algorithm was used to identify potential small molecule binding sites on the protein (Kawabata, 2010), and a docking pocket with volume 2,571 Å^3^ was defined. UCSF DOCK 6.9 was used for virtual screening and molecular docking. Finally, PyMol was used to visualise the docked conformation, and Ligplus was used to analyse the interaction force (Wallace et al., 1995).

## Drug Sensitive Analysis

The 23,808 identified RNA expression data and 23,255 analyzed drug data from the NCI-60 cell line were downloaded from the CellMiner database (Reinhold et al., 2012). To ensure the clinical practicality of the analysis results, FDA-approved drugs that had undergone clinical trial were selected to obtain a total of 792 drugs for the screening. MAP1LC3C expression was extracted and drug sensitivity (IC50) was calculated to obtain Pearson correlation coefficients between gene expression and different drugs, and the results were screened and visualized according to *p* < 0.05.

## Results

### Differential Expression of MAP1LC3C in 33 Cancer Types

**FIGURE 1:**
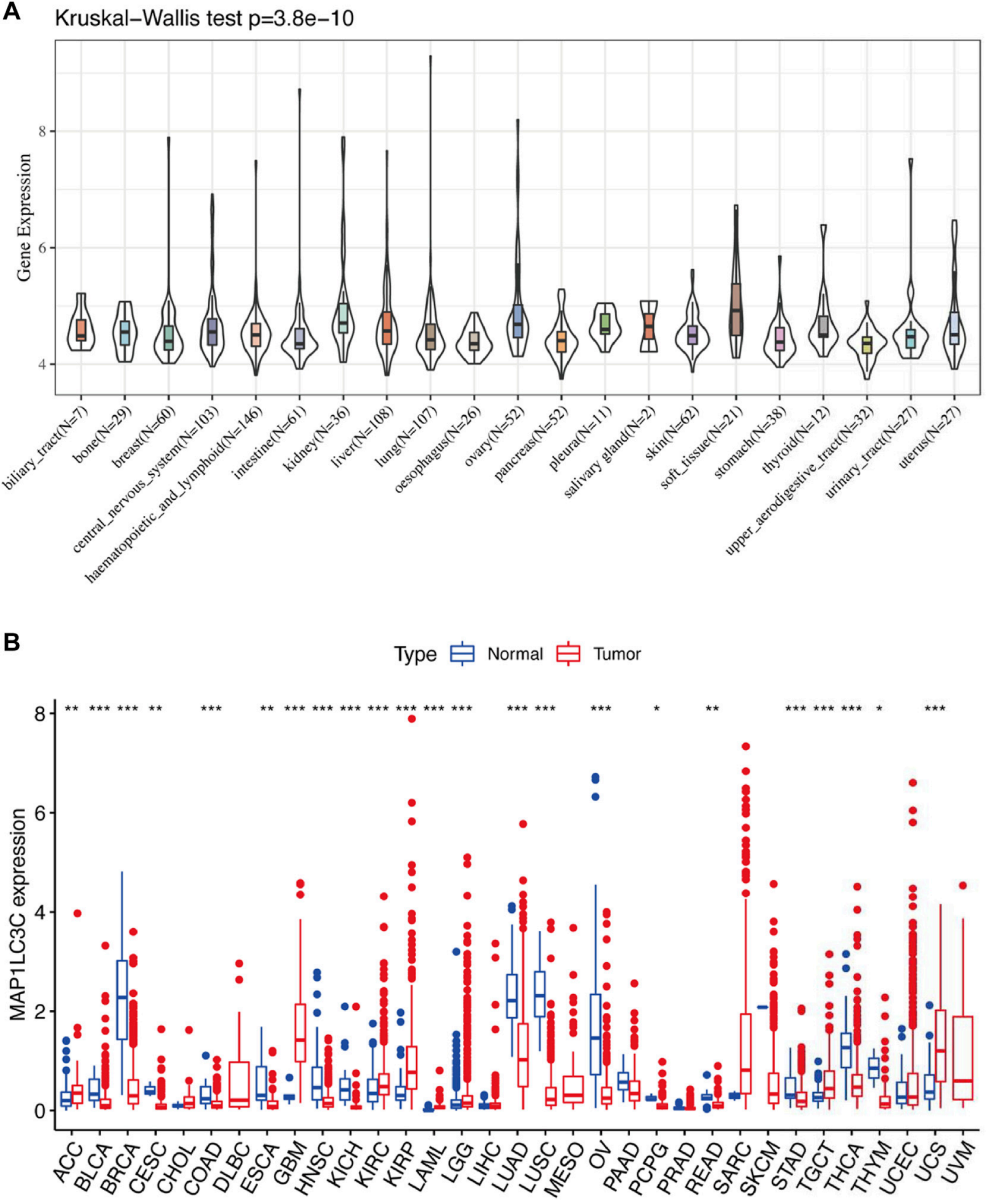
Human pan-cancer analysis of MAP1LC3C expression level. **(A)** mRNA level of MAP1LC3C in the CCLE database. **(B)** mRNA level of MAP1LC3C in the TCGA and GTEx database (*p < 0.05; **p <0.01; ***p < 0.001).

**FIGURE 2:**
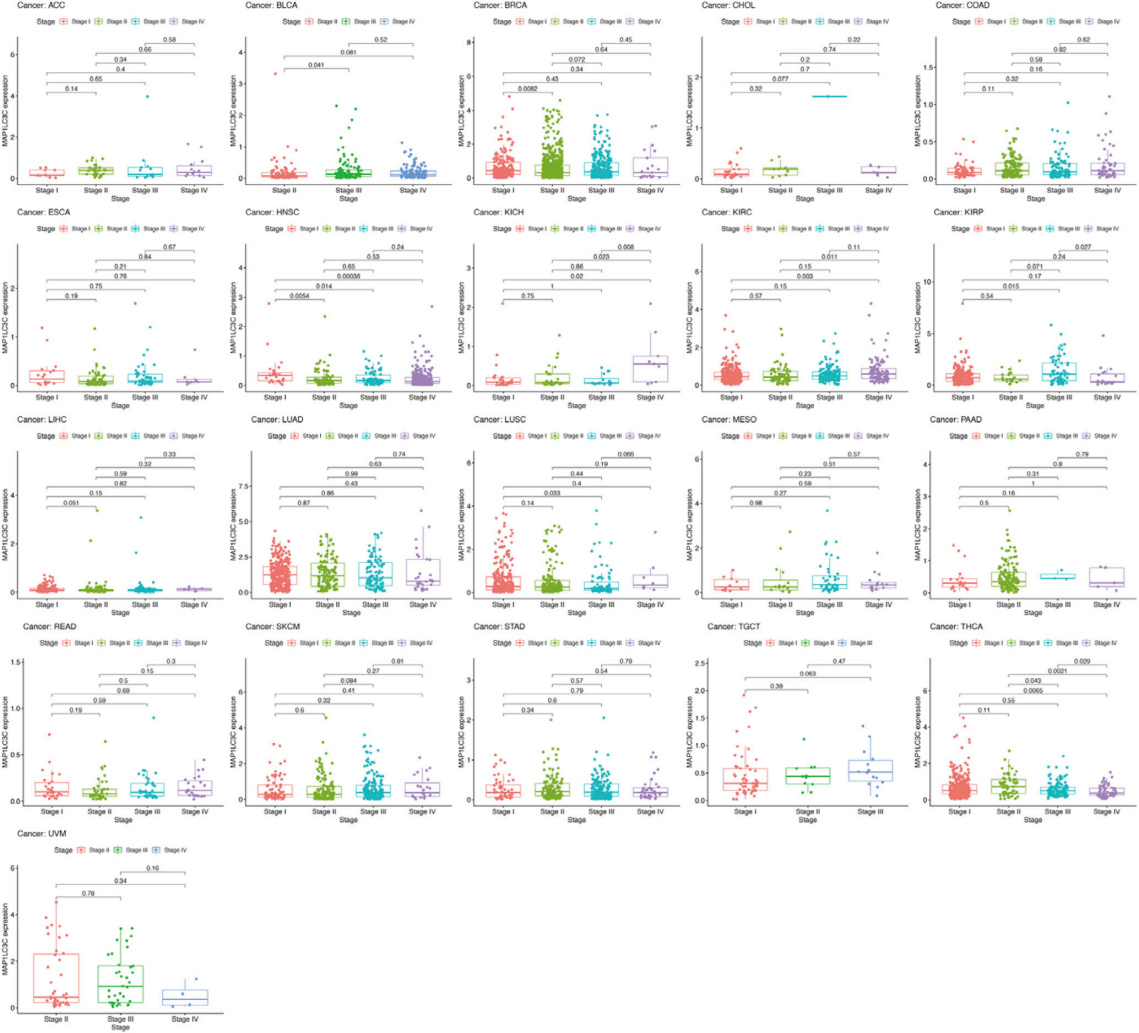
Boxplot graph indicating the relationship between MAP1LC3C expression and pathological stage in 21 cancer types.

**FIGURE 3:**
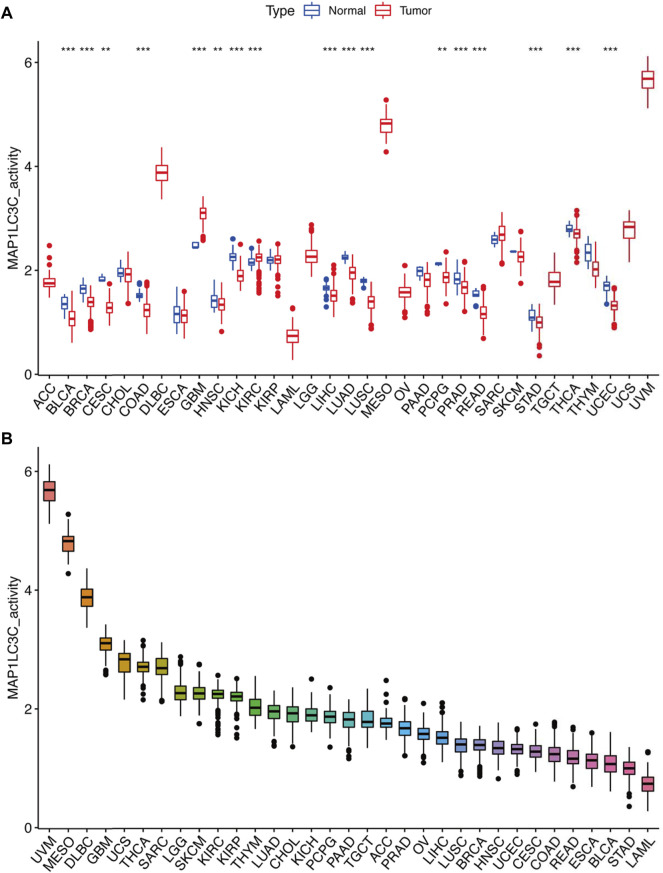
Calculation and investigation of MAP1LC3C activity. **(A)** The activities of MAP1LC3C in normal and tumour tissues in 33 cancer types. **(B)** The average activity (from high to low) of MAP1LC3C (*p < 0.05; * *p < 0.01; * * *p < 0.001).

Detailed information on the 33 types of cancer included in this study is shown in Supplementary Table S2. The gene expression profiles of various tumour cell lines downloaded from the CCLE database, and the MAP1LC3C expression levels of 21 tissues according to their tissue sources were analyzed. MAP1LC3C showed inconsistent expression levels in different cell lines (*p* = 3.8E-10, Figure 1A), but in soft tissue tumour cells showed the highest expression levels. Considering that several tumours in the TCGA database do not include normal sample data, normal sample data from the GTEx database were integrated with the tumour sample data from the TCGA database to analyse the expression differences of MAP1LC3C in 33 tumour types (Figure 1B). MAP1LC3C was significantly up-regulated in ACC, GBM, KIRC, KIRP, LAML, LGG, TGCT and UCS, but significantly down-regulated in BLCA, BRCA, CESC, COAD, ESCA, HNSC, KICH, LUAD, LUSC, OV, PCPG, READ, STAD, THCA and THYM. Meanwhile, MAP1LC3C expression was significantly correlated with the neoplasm staging of a few cancers, including HNSC, KICH, KIRC and THCA (Figure 2). As shown in Figure 3, TCGA data were used to analyse MAP1LC3C activity in 33 tumour types. The results showed that the transcription level was matched with MAP1LC3C activity. MAP1LC3C activity increased significantly in GBM, KIRC, BLCA, BRCA, CESC, COAD, HNSC, KICH, LIHC and LUAD tumour types, but decreased significantly in LUSC, PCPG, PRAD, READ, STAD, THCA and UCEC tumour types (Figure 3A). 4 tumour types including UVM, MESO, DLBC and GBM showed relatively high activity (Figure 3B).

## Prognostic Role of MAP1LC3C Expression

**FIGURE 4:**
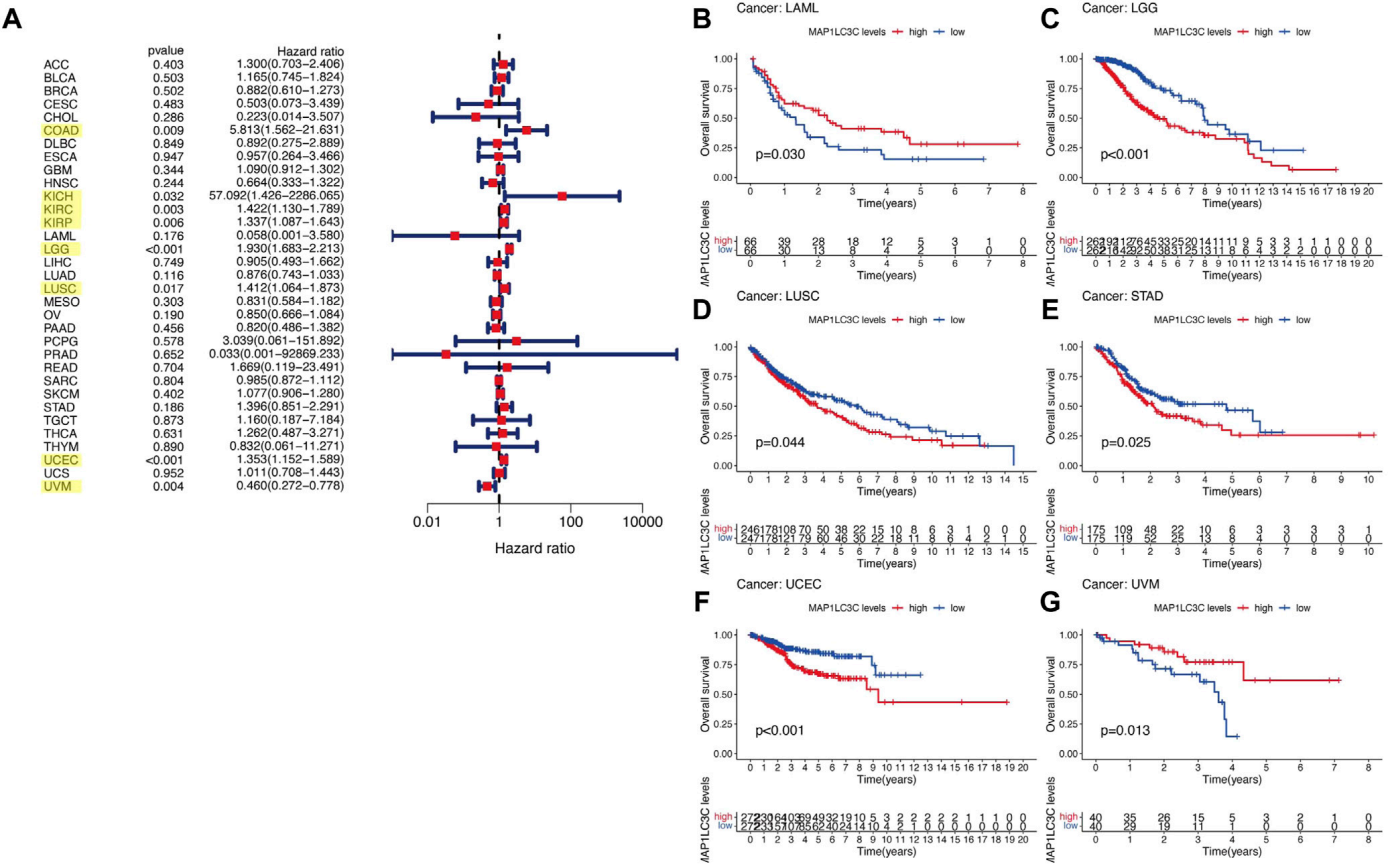
Relationship between MAP1LC3C expression and patients’ overall survival (OS) using **(A)** Forest map and **(B–G)** Kaplan-Meier analysis. The highlighted items mean that MAP1LC3C expression was significantly associated with the prognosis in these types of cancer (*p* < 0.05).

**FIGURE 5:**
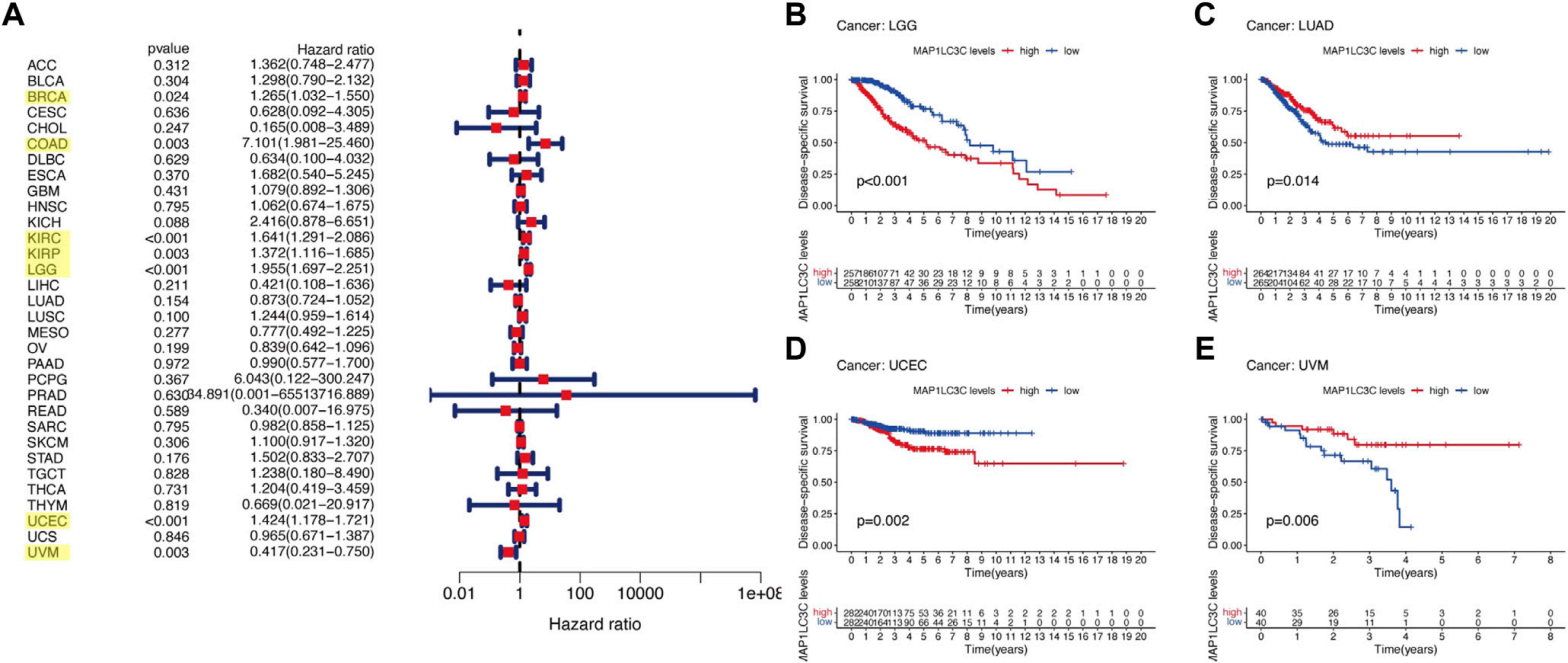
Relationship between MAP1LC3C expression and patients’ disease-specific survival (DSS) using **(A)** Forest map and **(B–E)** Kaplan-Meier analysis. The highlighted items mean that MAP1LC3C expression was significantly associated with the prognosis in these types of cancer (*p* < 0.05).

**FIGURE 6:**
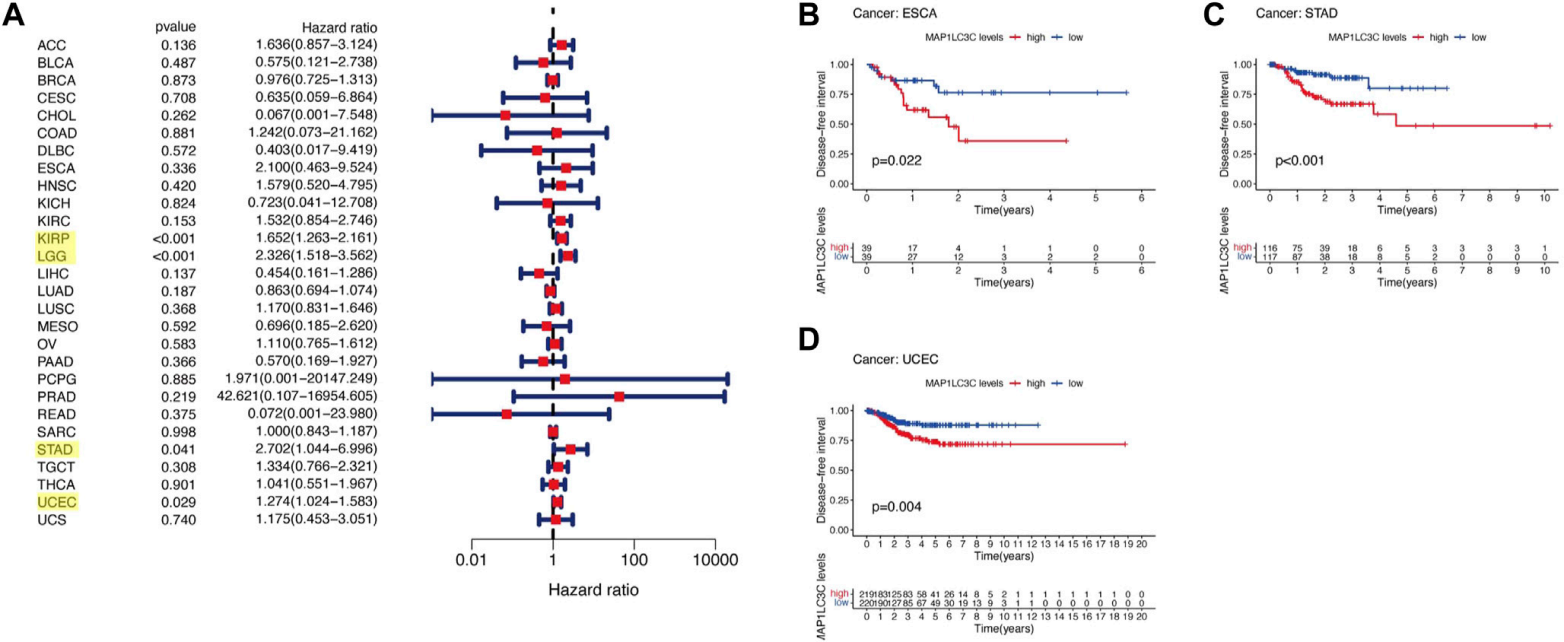
Relationship between MAP1LC3C expression and patients’ disease-free interval (DFI) using **(A)** Forest map and **(B–D)** Kaplan-Meier analysis. The highlighted items mean that MAP1LC3C expression was significantly associated with the prognosis in these types of cancer (*p* < 0.05).

**FIGURE 7:**
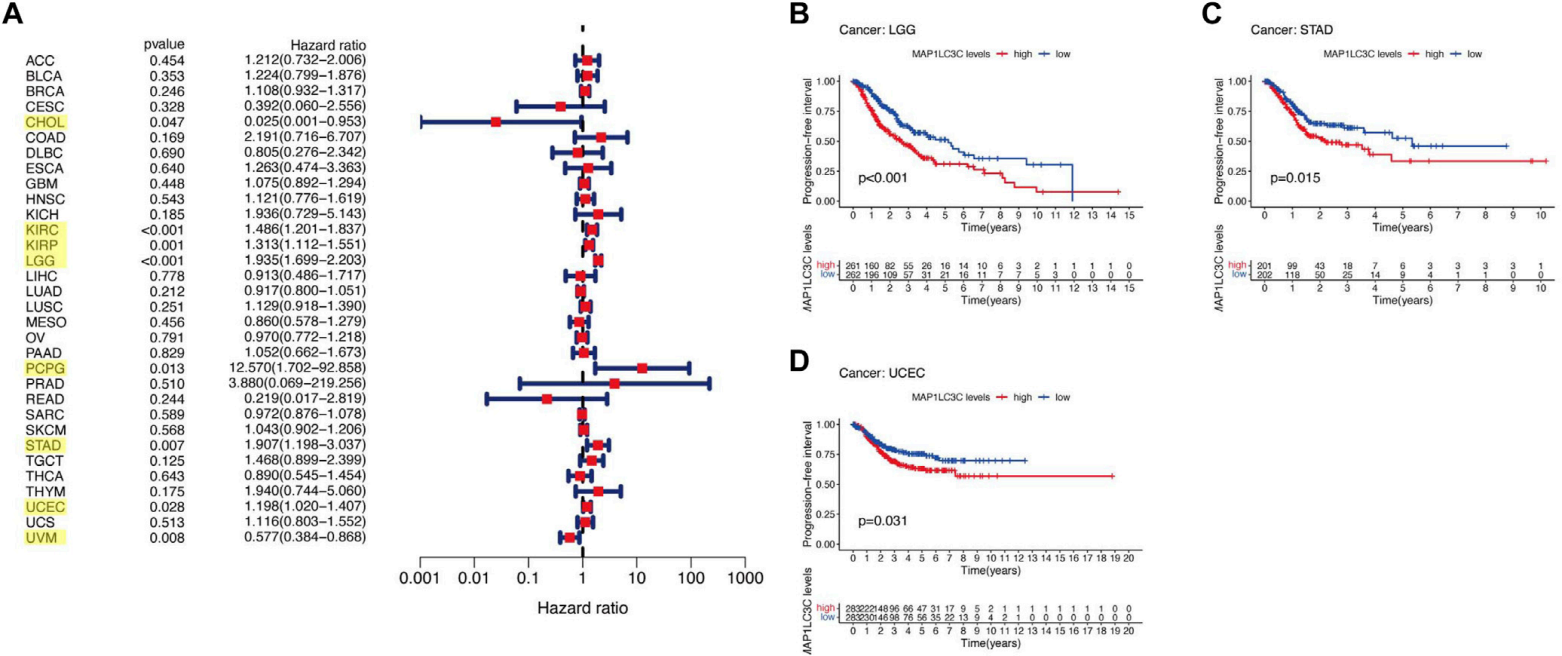
Relationship between MAP1LC3C expression and patients’ progression-free interval (PFI) using **(A)** Forest map and **(B–D)** Kaplan-Meier analysis. The highlighted items mean that MAP1LC3C expression was significantly associated with the prognosis in these types of cancer (*p* < 0.05).

For the prognostic analysis, we selected clinical indicators, including overall survival (OS), disease-specific survival (DSS), disease-free interval (DFI) and progression-free interval (PFI). OS was defined as the time from the date of diagnosis to death, regardless of the cause. In OS analysis, Univariate Cox regression identified high MAP1LC3C expression as a risk factor for COAD, KICH, KIRC, KIRP, LGG, LUSC and UCEC, but as a protective factor for UVM (Figure 4A). KM analysis revealed that patients with high MAP1LC3C expression in LGG, LUSC, STAD and UCEC cancer types had lower OS rates than those with low MAP1LC3C expression. However, those with high MAP1LC3C expression in LAML and UVM had higher OS rates (Figures 4B–G). In DSS analysis, unlike OS, patients who died from causes other than the specified disease were not counted. Univariate Cox regression indicated high MAP1LC3C expression as a risk factor for BRCA, COAD, KIRC, KIRP, LGG and UCEC, but as a protective factor for UVM (Figure 5A). KM analysis demonstrated that patients with high MAP1LC3C expression in LGG and UCEC had lower DSS rates than those with low MAP1LC3C expression, while those with high MAP1LC3C expression in LUAD and UVM had higher DSS rates (Figures 5B–E). In DFI analysis, patients who died from causes other than the specified disease were not counted. Univariate Cox regression identified high MAP1LC3C expression as a risk factor for KIRP, LGG, STAD and UCEC (Figure 6A). KM analysis showed that patients with high MAP1LC3C expression in ESCA, STAD and UCEC had lower DFI rates than those with low MAP1LC3C expression (Figures 6B–D). Unlike DFI, PFI was defined as progression or death from disease, again from any cause. In PFI analysis, Univariate Cox regression identified high MAP1LC3C expression as a risk factor for KIRC, KIRP, LGG, PCPG, STAD and UCEC, but as a protective factor for CHOL and UVM (Figure 7A). KM analysis confirmed that patients with high MAP1LC3C expression in LGG, STAD and UCEC had lower PFI rates than those with low MAP1LC3C expression (Figures 7B–D). Notably, KIRP, LGG and UCEC showed significant differences in all four analyses above, and high MAP1LC3C expression suggested a poor prognosis in all three types of cancers.

### Correlation Between MAP1LC3C Expression and Tumour Immunity

**FIGURE 8:**
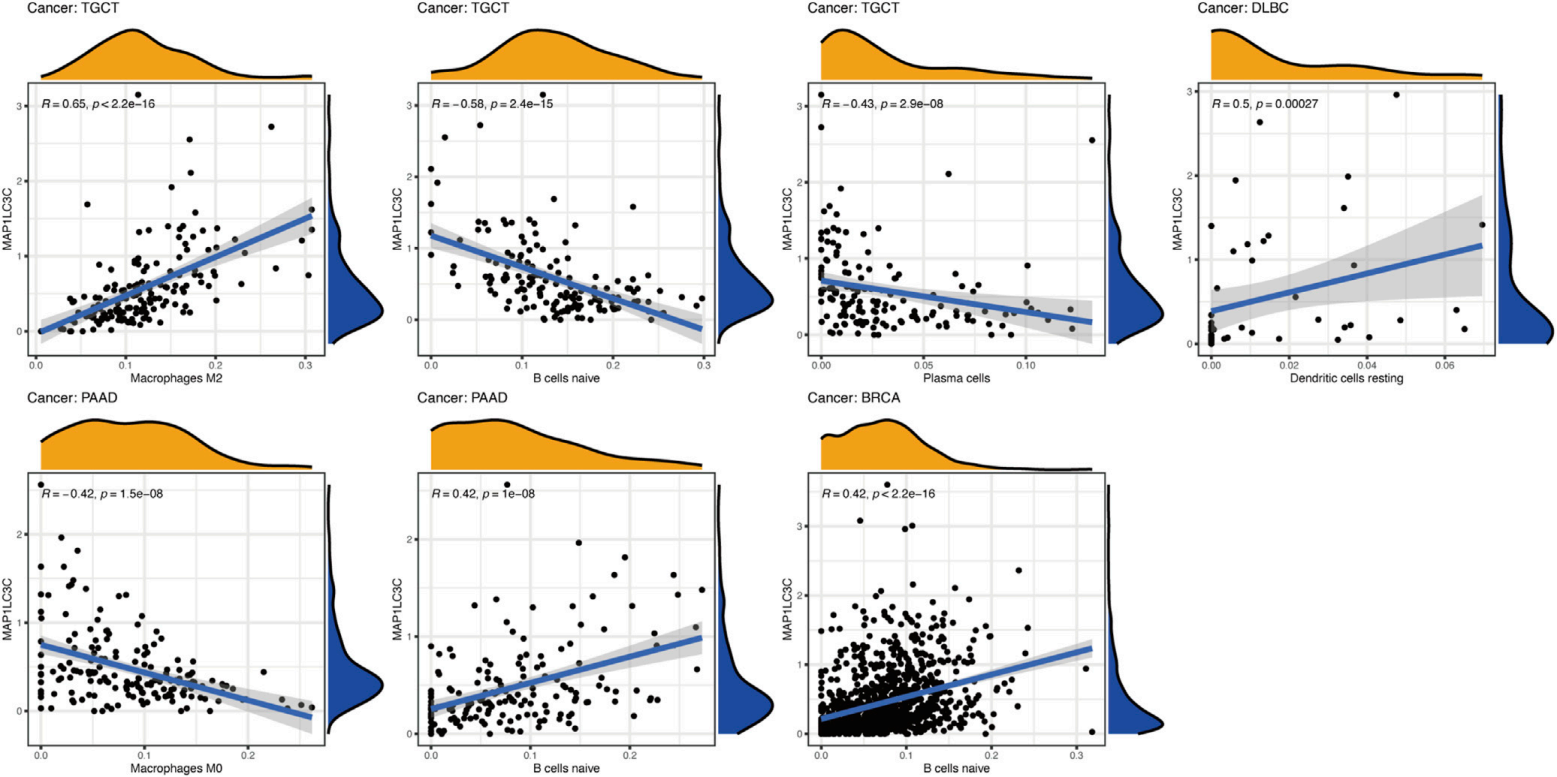
Correlation analysis between MAP1LC3C expression and immune cell infiltration using CIBERSORT algorithm.

**FIGURE 9:**
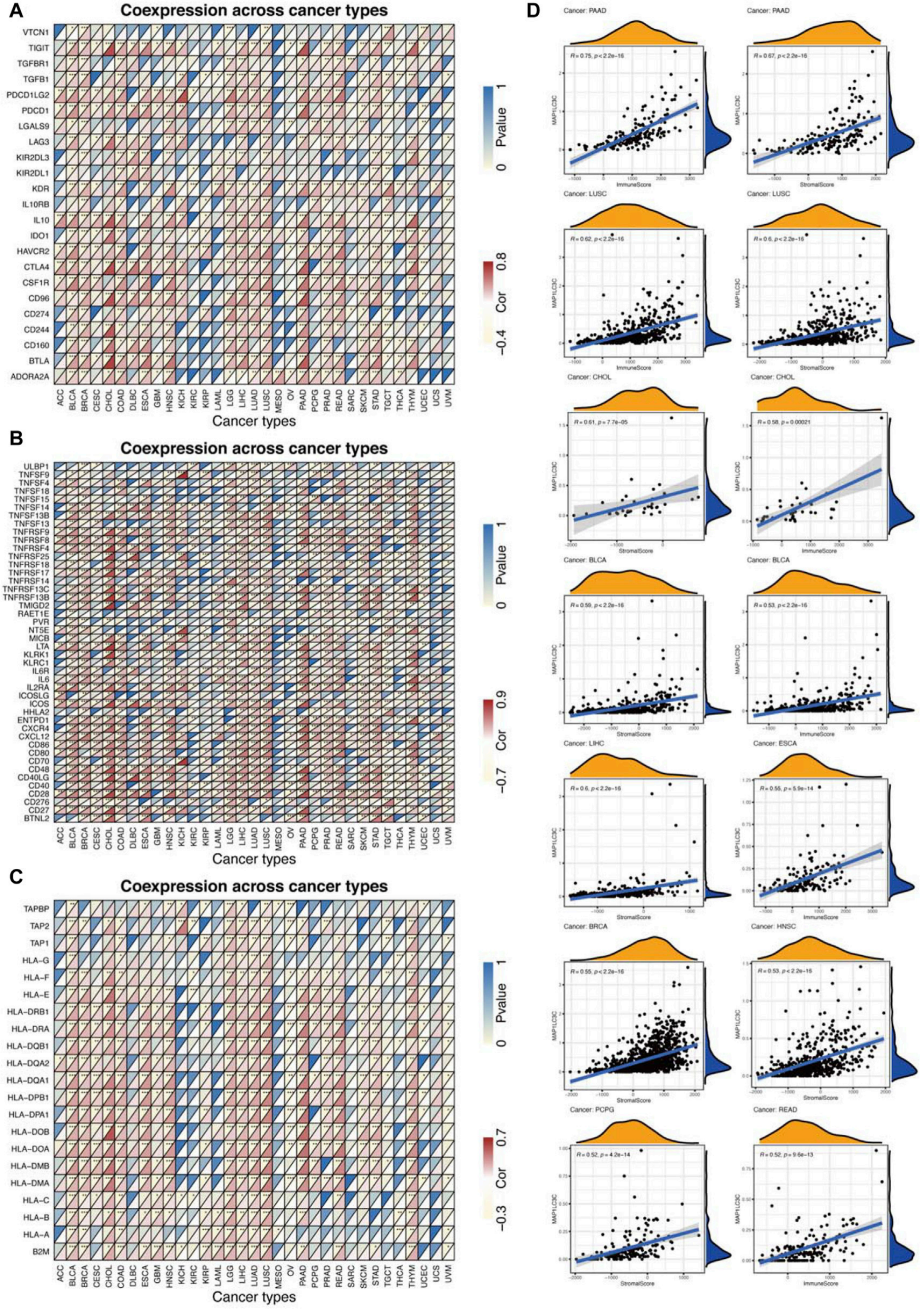
Relationship between MAP1LC3C expression and cancer immunity. **(A)** Heat maps of the correlation between MAP1LC3C expression and immune inhibitors, **(B)** immune stimulators and **(C)** major histocompatibility complex (MHC) molecules in 33 cancer types. For each grid in Figures **(A–C)**, the colour of the upper left triangle represents the *p*-value, and the colour of the lower right triangle represents the Spearman correlation coefficient (*p < 0.05; **p < 0.01; ***p < 0.001). **(D)** Correlation analysis between MAP1LC3C expression and ESTIMATE scores in 33 cancer types.

Using CIBERSORT, the detailed immune cell composition of all patients in the TCGA database was calculated, and the correlation between 22 immune cells in 33 cancer types and MAP1LC3C expression was determined (Supplementary Table S3). The results revealed that a majority of immune cells were significantly correlated with MAP1LC3C expression. As shown in Figure 8, three types of immune cells in TGCT, two types in PAAD and one type in DLBC and BRCA were correlated with the expression of MAP1LC3C, respectively (*p* < 0.01 and |R| > 0.4). As shown in Figure 9A, 23 immune inhibitors were analyzed, and the expression of MAP1LC3C was significantly correlated with the immune inhibitors of various cancer types, including CHOL, LGG and PAAD. Correlation analysis of 45 immune stimulators revealed a significantly positive correlation between MAP1LC3C expression in CHOL and PAAD and many immune stimulators in Figure 9B. Additionally, correlation analysis of 21 MHCs revealed a significantly positive correlation between MAP1LC3C expression in CHOL, LGG, LIHC and LUSC and multiple MHC molecules in Figure 9C.

ESTIMATE algorithm was used to calculate the tumour tissue’s immuno/stromal-scores and evaluate the relationship between the immuno/stromal-scores and MAP1LC3C expression. Figure 9D showed a significantly positive correlation of MAP1LC3C expression in PAAD, LUSC, CHOL, BLCA, LIHC, ESCA, BRCA, HNSC, PCPG and READ with immuno/stromal-scores (*p* < 0.01 and |R| > 0.5). The detailed results of ESTIMATE scores are summarized in Supplementary Table S4.

**FIGURE 10:**
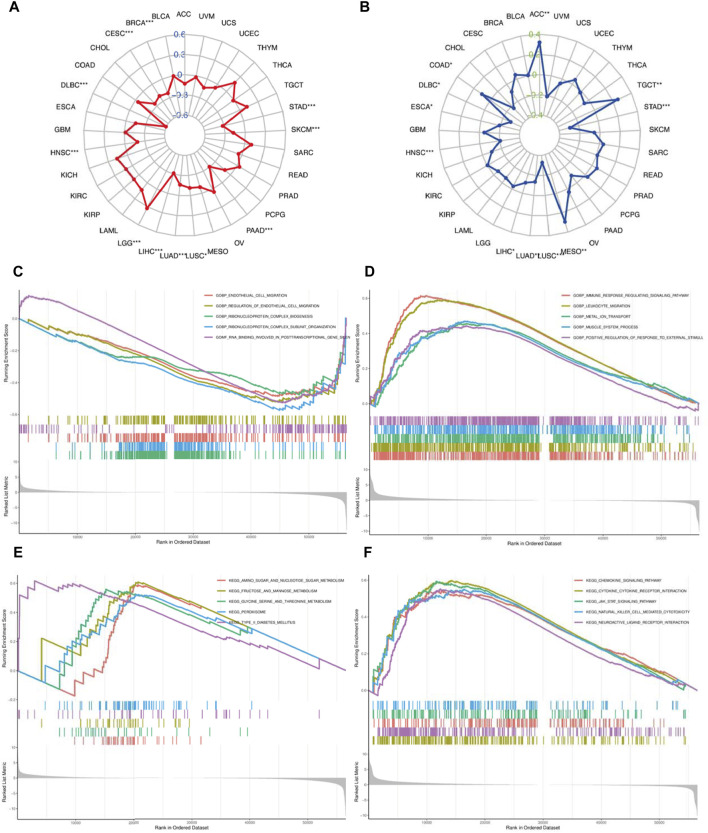
**(A)** Correlation of MAP1LC3C expression with tumour mutational burden (TMB) and **(B)** microsatellite instability (MSI). The blue and green number represent Spearman’s correlation coefficient for TMB and MSI, respectively (*p < 0.05; * *p < 0.01; ***p <0.001). GSEA analysis shows the GO terms related to MAP1LC3C expression in **(C)** KIRC and **(D)** LGG. GSEA analysis shows the KEGG pathways associated with MAP1LC3C expression in **(E)** KIRC and **(F)** LGG.

The correlation between TMB/MSI and MAP1LC3C expression (Supplementary Table S5, Figures 10A,B) were evaluated. As shown in Figure 10A, only LGG showed a significantly positive correlation between MAP1LC3C expression and TMB, whereas STAD, SKCM, PAAD, LUSC, LUAD, LIHC, HNSC, DLBC, CESC and BRCA showed a significantly negative correlation. Moreover, MAP1LC3C levels were significantly positively correlated with MSI in ACC, COAD, MESO and TGCT, but significantly negatively correlated with DLBC, ESCA, HNSC, LIHC, LUAD, LUSC and STAD (Figure 10B). Therefore, MAP1LC3C expression is closely associated with the tumour immune microenvironment in a variety of cancers.

## Biological Function of MAP1LC3C

**FIGURE 11:**
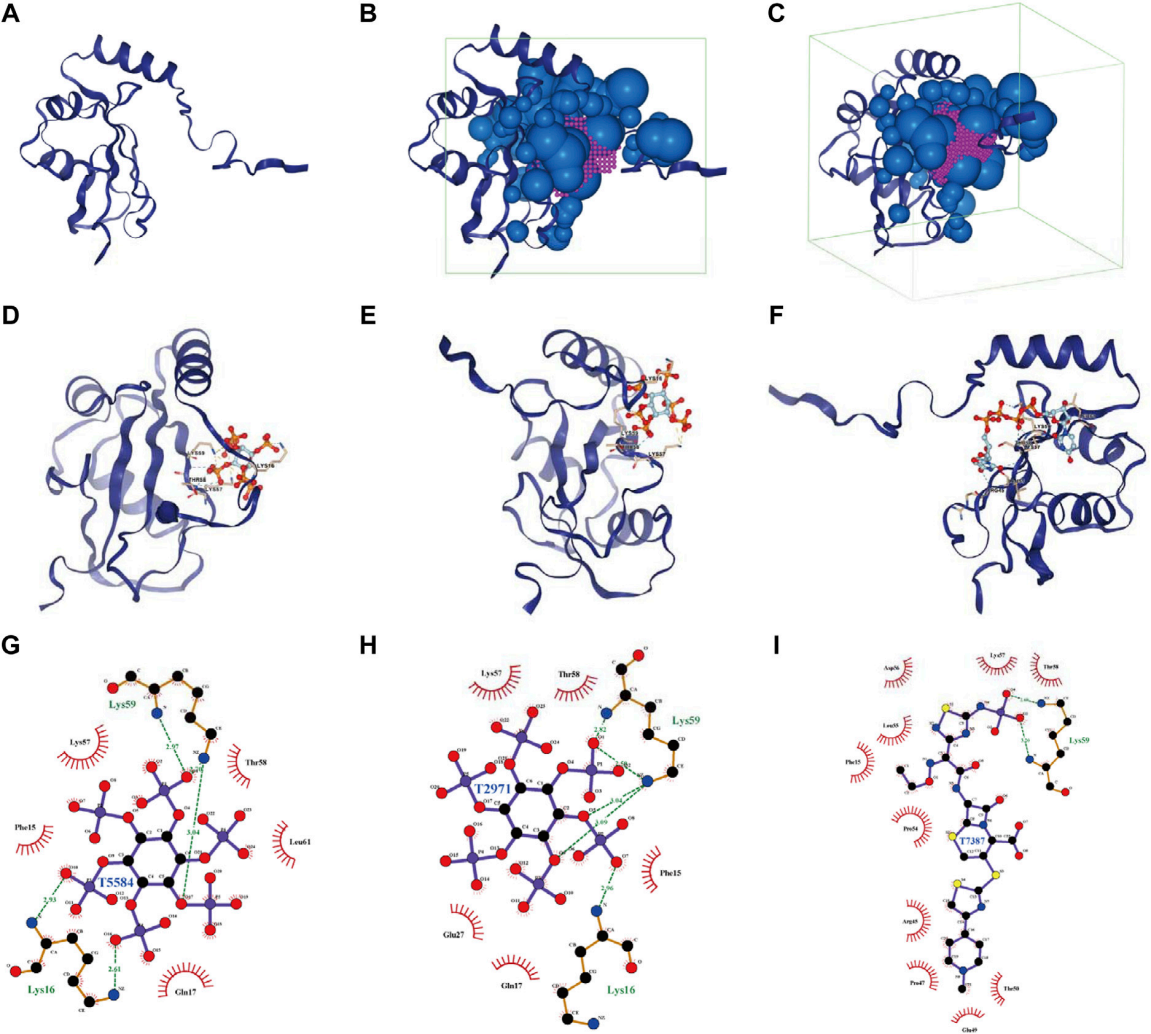
Analysis of docking conformation and interaction force between MAP1LC3C and FDA-approved and pharmacopeia drugs obtained *via* virtual screening. **(A)** The three-dimensional structure of the MAP1LC3C protein. **(B, C)** The binding site and box of MAP1LC3C protein (purple, binding site; green, binding box; blue, little ball). **(D–F)** The three-dimensional docking conformations of T5584, T2971 and T7387 with MAP1LC3C visualised using PyMOL (Yellow dotted line, salt bridge (ionic bond); blue dotted line, hydrogen bond; residues are identified in the form of labels). **(G–I)** Ligplus shows a two-dimensional analysis of the drug molecule (middle)’s interaction forces with the associated amino acid residues (green dotted lines, hydrogen bonds; green amino acid names, amino acid residues forming the hydrogen bonds).

Given the differential expression of MAP1LC3C in many cancers, GSEA was used to assess the biological function of MAP1LC3C in 33 cancer types. In KIRC, MAP1LC3C shows enrichment in the following GO terms: GOBP_ENDOTHELIAL_CELL_MIGRATION, GOBP_REGULATION_OF_ENDOTHELIAL_CELL_MIGRATION, GOBP_RIBONUCLEOPROTEIN_COMPLEX_BIOGENESIS, GOBP_RIBONUCLEOPROTEIN_COMPLEX_BIOGENESIS and GOMF_RNA_BINDING_INVOLVED_IN_POSTTRANSCRIPTIONAL_GENE_SILENCING, and in the following KEGG terms: KEGG_AMINO_SUGAR_AND_NUCLEOTIDE_SUGAR_METABOLISM, KEGG_FRUCTOSE_AND_MANNOSE_METABOLISM, KEGG_GLYCINE_SERINE_AND_THREONINE_METABOLISM, KEGG_PEROXISOME and KEGG_TYPE_II_DIABETES_MELLITUS (Figures 10C,E). Additionally, in LGG, MAP1LC3C shows enrichment in the following GO terms: GOBP_IMMUNE_RESPONSE_REGULATING_SIGNALING_PATHWAY,GOBP_LEUKOCYTE_MIGRATION, GOBP_METAL_ION_TRANSPORT, GOBP_MUSCLE_SYSTEM_PROCESS and GOBP_POSITIVE_REGULATION_OF_RESPONSE_TO_EXTERNAL_STIMULUS, and in the following KEGG terms: KEGG_CHEMOKINE_SIGNALING_PATHWAY, KEGG_CYTOKINE_CYTOKINE_RECEPTOR_INTERACTION, KEGG_JAK_STAT_SIGNALING_PATHWAY, KEGG_NATURAL_KILLER_CELL_MEDIATED_CYTOTOXICITY and KEGG_NEUROACTIVE_LIGAND_RECEPTOR_INTERACTION (Figures 10D, 11F).

## Virtual Screening and Molecular Docking Based on MAP1LC3C Structure

Using virtual screening and molecular docking of the FDA-Approved and Pharmacopeia Drug Library, the top 10 drugs with the best docking score were obtained (Table 1). The complete drug docking scores are shown in Supplementary Table S6, all drug IDs with drug names are listed in Supplementary Table S7. Figure 11A shows the MAP1LC3C protein, and Figures 11B,C show the binding sites and boxes of this protein, respectively. Figures 11D–I show the docking conformation and interaction force analysis of the top three best-combined drugs (T5584 [sodium phytate hydrate], T2971 [phytic acid] and T7387 [ceftaroline fosamil]). The function of sodium phytate is as a [PO4]3- storage depot and precursor for other inositol phosphates and pyrophosphates. While *in vitro*, it is an effective chelator of divalent and trivalent cations (Shears, 2001). Phytic acid is being investigated in clinical trials NCT01000233 for its value in the prevention of cardiovascular calcifications. Ceftaroline fosamil is a cephalosporin antibacterial agent for the treatment of the following infections caused by specified susceptible bacteria: Acute bacterial skin and skin structure infections and community-acquired bacterial pneumonia (Steed and Rybak, 2010). In summary, all of these drugs play an important role in the treatment of non-oncological diseases, but our studies suggest that they also have potential therapeutic value in oncology.

**TABLE 1:** Top 10 drugs with the best docking score.

ID	Grid_Score	Grid_vdw_energy	Grid_es_energy	Internal_energy_repulsive
T5584	−138.6127	−37.7842	−100.8285	15.337
T2971	−138.3417	−32.2102	−106.1315	15.8525
T7423	−119.3529	−76.3458	−43.0071	19.8214
T7387	−107.2824	−61.3152	−45.9672	11.3924
T5036	−105.8314	−72.7612	−33.0702	9.9596
T5067	−101.6643	−76.9384	−24.726	19.2636
T2119	−99.7468	−80.3021	−19.4447	22.2605
T0313	−99.2818	−60.4463	−38.8356	44.4261
T5725	−98.9003	−73.7259	−25.1744	12.8408
T1352	−96.987	−54.7152	−42.2719	6.9319

### Correlation Between MAP1LC3C Expression and Drug Sensitivity

**FIGURE 12:**
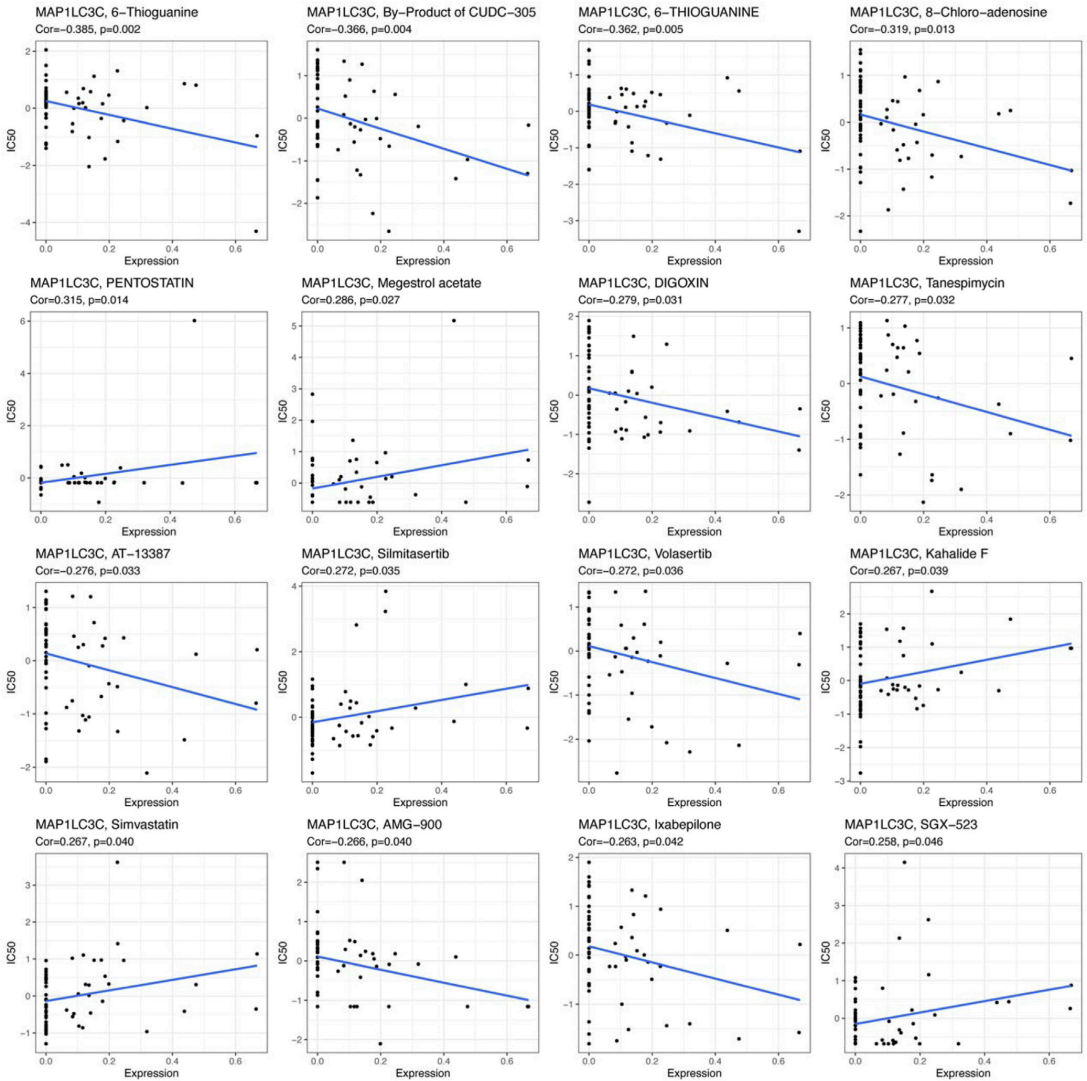
Scatter plot of the relationship between MAP1LC3C expression and drug sensitivity.

**FIGURE 13:**
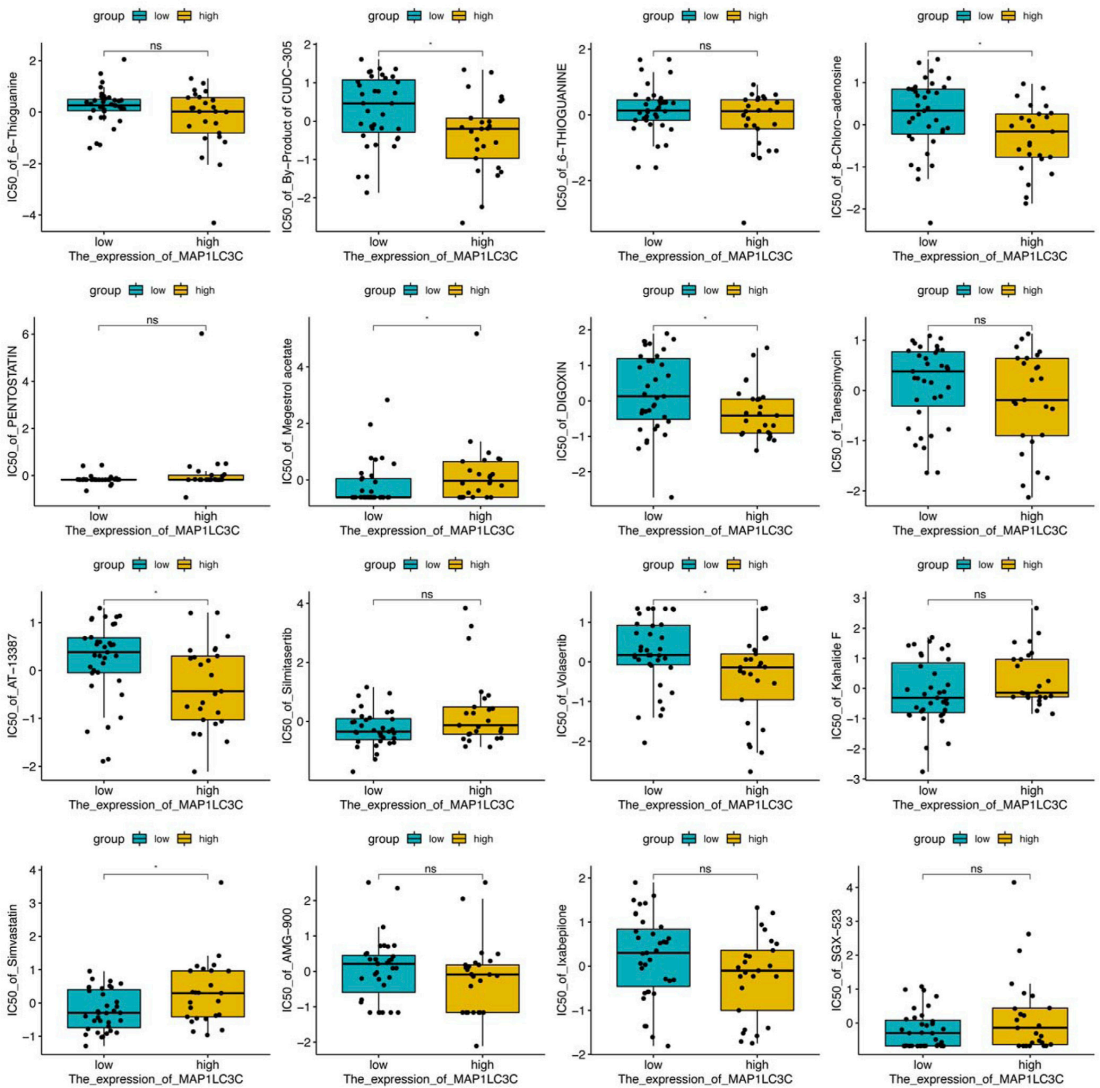
Boxplot of drug sensitivity in high and low expression groups of MAP1LC3C (*p < 0.05).

The examination of MAP1LC3C expression in NCI-60 cell lines showed a significant correlation between MAP1LC3C expression and drug sensitivity (Table 2). In particular, as MAP1LC3C expression increased, 6-Thioguanine, By-Product of CUDC-305, 8-Chloro- adenosine, DIGOXIN, AT-13387 and Volasertib had a lower IC50 against cancer cells (Figures 12, 13). This implies that increased expression of MAP1LC3C enhanced the sensitivity of cancer cells to these drugs. It is notable that Volasertib, which showed better results in the virtual screening, also obtained better results in the drug sensitivity analysis.

**TABLE 2:** Correlation of MAP1LC3C expression with FDA-approved/clinical trial drug sensitivity.

Gene	Drug	Cor	*p*-value
MAP1LC3C	6-Thioguanine	−0.3,852,155	0.00237,106
MAP1LC3C	By-Product of CUDC-305	−0.366,031	0.00402,544
MAP1LC3C	6-THIOGUANINE	−0.3,616,411	0.00452,394
MAP1LC3C	8-Chloro-adenosine	−0.3,187,263	0.01,306,425
MAP1LC3C	PENTOSTATIN	0.31,547,869	0.01,407,516
MAP1LC3C	Megestrol acetate	0.28,630,048	0.0265,773
MAP1LC3C	DIGOXIN	−0.2,790,328	0.03,085,066
MAP1LC3C	Tanespimycin	−0.2,772,625	0.03,197,435
MAP1LC3C	AT-13387	−0.2,762,148	0.03,265,537
MAP1LC3C	Silmitasertib	0.27,230,956	0.03,530,124
MAP1LC3C	Volasertib	−0.2,715,721	0.03,582,036
MAP1LC3C	Kahalide F	0.26,665,574	0.03,944,544
MAP1LC3C	Simvastatin	0.26,650,703	0.03,955,965
MAP1LC3C	AMG-900	−0.2,659,905	0.03,995,851
MAP1LC3C	Ixabepilone	−0.2,630,051	0.04,232,913
MAP1LC3C	SGX-523	0.25,809,477	0.04,647,964
MAP1LC3C	XAV-939	0.25,738,989	0.04,710,194

## Discussion

This study aimed to comprehensively investigate the differential expression of MAP1LC3C between the normal and tumour tissues and to study the prognostic effect and potential immunotherapeutic value of MAP1LC3C for various tumours. The results indicate that MAP1LC3C expression varies in different cancer types. Moreover, survival analysis revealed that abnormal MAP1LC3C expression plays a prognostic role in various types of cancer, including BRCA, COAD, ESCA, KICH, KIRC, KIRP, LAML, LGG, LUAD, LUSC, PCPG and STAD. Additionally, MAP1LC3C expression was correlated highly with the tumour microenvironment, immune cells, immune modulators, TMB and MSI. The biological functions and signalling pathways related to MAP1LC3C expression were also identified. Finally, FDA-approved drugs were screened through virtual screening and drug sensitivity analysis.

Immunotherapy represents a shift in treatment modalities for oncology, the goal is no longer to target the tumour itself, but to overcome the immune suppression caused by the tumour and its microenvironment, and then allow the immune system to target and kill the cancer cells (Billan et al., 2020). About 10 years ago, the first immune checkpoint inhibitor, ipilimumab, a monoclonal antibody targeting CTLA-4, was approved for the FDA and it marked the beginning of the immunotherapy era (Squibb, 2020a). Two additional immune checkpoint inhibitors targeting PD-1 (pembrolizumab and nivolumab) were subsequently approved, and all three were initially approved for unresectable or metastatic melanoma (Squibb, 2020b; Merck, 2020). More immune checkpoint inhibitors have subsequently been approved for a variety of cancers. As a result, immunotherapy is increasingly being used in the clinical management of tumours and our study also focuses on the analysis of the value of MAP1LC3C in immunotherapy to provide new insights into future immunotherapy regimens.

Identifying abnormally expressed genes and tumour-specific targets or features for personalized treatment in different cancers could increase the possibility of cure or remission for patients (Andre et al., 2014). Therefore, the use of TCGA and GTEx databases for pan-cancer analysis helps to determine the differential expression and role of MAP1LC3C in various types of cancer (Cao and Zhang, 2016; Cava et al., 2018). Furthermore, a thorough pan-cancer analysis can be performed in cell lines using the CCLE database to assess gene expression, which may have implications for future cell experiments. Consistent with previously reported results, differentially expressed MAP1LC3C has certain prognostic values in some cancers, especially COAD, LGG and LUAD. MAP1LC3C is a marker that indicates poor prognosis in patients with colon cancer, low-grade glioma and lung adenocarcinoma (Xu et al., 2020; Guo et al., 2021; Wang et al., 2021).

Generally, the protein expression level better reflects the tissue activity (Mo et al., 2021). Due to the lack of relevant data on protein expression levels in public databases, it is difficult to study MAP1LC3C protein expression level. However, MAP1LC3C activity score in various cancer types was obtained using the ssGSEA method. By comparing the transcription level and activity score, the transcription levels of some cancers (BLCA, BRCA, CESC, COAD, GBM, HNSC, KICH, KIRC, LUAD, LUSC, PCPG, READ, STAD and THCA) were matched with MAP1LC3C activity, indicating that the transcription levels represented activation or repression of MAP1LC3C. If the transcription levels could not be matched with MAP1LC3C activity in certain cancers, it indicates that it may be due to post-transcriptional protein level modifications or protein metabolism, which affect MAP1LC3C expression. In our study, there were no cancers with mismatches.

Tumour tissue contains not only tumour cells but also immune cells. Immune cells that infiltrate the tumours can profoundly influence tumour development and anti-cancer therapy (Dalton et al., 1993). Therefore, the quantification of immune cells has extraordinary significance. Recently, immunotherapy has shown an increased efficacy in the treatment of tumours (Finn, 2008). This study reports that the expression level of MAP1LC3C is related to cancer immunity. A strong correlation between MAP1LC3C and macrophages M2, B cells naive and plasma cells was observed in TGCT. In PAAD, a strong correlation was noted between MAP1LC3C and macrophages M0 and B cells naive. Correlation analysis of 23 immune inhibitors showed that MAP1LC3C expression was significantly correlated with various immune inhibitors agents of different cancer types, especially CHOL, LGG and PAAD. Correlation analysis of 45 immune activators showed a significantly positive correlation of MAP1LC3C expression in CHOL and PAAD with many immune activators. The correlation between MAP1LC3C expression and 21 MHCs was also analysed. Human leukocyte antigen (HLA) is the expression product of human MHC, which is the most complex polymorphic system in the human body (Norman et al., 2017). It is worth noting that MHC is closely related to human immune response, immune regulation and few pathological state generation (Xu et al., 2006; Wang et al., 2013). The results indicated that most cancers were positively correlated with HLA. ESTIMATE is a tool for analysing tumour purity and stromal and immune cells’ presence in the tumour (Yoshihara et al., 2013). ESTIMATE algorithm generates four final scores: stromal score (indicating the presence of stromal cells in the tumour tissue), immune score (indicating the invasion of immune cells in the tumour tissue), ESTIMATE score and tumour purity score. Our results revealed that MAP1LC3C expression in PAAD, LUSC, CHOL and BLCA had a significantly positive correlation with matrix fraction and immune fraction. In ESCA and READ, MAP1LC3C expression was significantly positively correlated with immune score, and in LIHC, BRCA, HNSC and PCPG, MAP1LC3C expression was significantly positively correlated with stromal scores.

Gene mutation is the main cause of cancer (Martincorena and Campbell, 2015). Specific gene mutations can be used to predict patient prognosis and treatment outcome (Sanz-Garcia et al., 2017). There is a likelihood that more neoantigens are formed with more somatic mutations in a tumour, which can help the adaptive immune system in recognising and detecting cancer. TMB provides a useful estimate of the tumour-neoantigen load (Chan et al., 2019), and the level of TMB affects the production of immunogenic peptides, thereby influencing the response of patients to immune checkpoint inhibitors (Havel et al., 2019). Additionally, MSI is a powerful mutant phenotype caused by DNA mismatch repair defects (Yamamoto and Imai, 2019), and it is an important indicator for predicting tumour occurrence and development (Li et al., 2020). MSI is also used as an FDA-approved biomarker for immunotherapy (Lemery et al., 2017). Our study reports that MAP1LC3C is highly negatively correlated with these two immunotherapy biomarkers (TMB and MSI) in a few cancers, such as DLBC, HNSC, LIHC, LUAD, LUSC and STAD. These results suggest that MAP1LC3C may influence the immunotherapy response of these six cancer types.

Furthermore, virtual screening is a commonly used computational technique for drug designing. Virtual screening can be divided into two categories: structure-based virtual screening and ligand-based virtual screening (Lavecchia and Di Giovanni, 2013; Forli, 2015). Virtual screening based on the three-dimensional structure of the receptor (target protein) was adopted to screen for drugs that had a good interaction with the MAP1LC3C protein from the FDA-Approved and Pharmacopeia Drug Library. Furthermore, a drug sensitivity analysis using data from the NCI-60 cell line was performed. On the one hand, these results demonstrate the feasibility of MAP1LC3C as a drug target and, on the other hand, provide a reference for the development of therapeutic drug regimens. Notably, the drug with better results in both the virtual screen and the drug sensitivity analysis is Volasertib, a Plk1 inhibitor, that has reached phase III clinical trials for adult acute myeloid leukaemia patients ineligible for intensive remission induction therapy (Döhner et al., 2016). Given the better results of Volasertib in both drug sensitivity analysis and molecular docking against MAP1LC3C, it is worth trying to explore its therapeutic value in other cancers. In general, all the screened drugs can be considered as potential therapeutic agents for various cancer types. However, further *in vivo* studies need to be conducted.

## Conclusion

To the best of our knowledge, this study is the first report to investigate the prognostic and therapeutic value of MAP1LC3C in 33 types of cancer. Our results suggested that MAP1LC3C can be a valuable prognostic biomarker for certain types of cancer, and showed high correlation with important immunological indexes in certain cancers. This will facilitate us to understand the role of MAP1LC3C in the immune system and lay a solid theoretical foundation for future immunotherapy. The FDA-approved drugs identified using virtual screening and drug sensitivity analysis could be potential cancer therapeutic agents, thereby paving the way for future cancer treatment research. The bioinformatics method was used in this study to provide relatively preliminary results and in future in-depth studies are needed to clarify the association between MAP1LC3C and cancer treatment.

## Data Availability

The datasets presented in this study can be found in online repositories. The names of the repository/repositories and accession number(s) can be found in the article/Supplementary Material.

## Finding

This project was sponsored by Liaoning Provincial Natural Science Foundation (2020-MS-155), China Medical University novel coronavirus pneumonia prevention and control research project (No. 2020-12-11), Scientific Research Foundation for the Returned Overseas Chinese Scholars, State Education Ministry (2013-1792), National Science Foundation of China (72074104), Shenyang Planning Foundation for Science and Technology (21-173-9-38), and the first batch of medical education scientific research project of China Medical University for the 14th Five-Year Plan (YDJK2021039). The researchers are grateful for the support of several organizations.

## References

[B1] Andre F. Mardis E. Salm M. Soria J. C. Siu L. L. Swanton C. (2014). Prioritizing Targets for Precision Cancer Medicine. Ann. Oncol. 25 (12), 2295–2303. 10.1093/annonc/mdu478 25344359

[B2] Berman H. M. Westbrook J. Feng Z. Gilliland G. Bhat T. N. Weissig H. (2000). The Protein Data Bank. Nucleic Acids Res. 28 (1), 235–242. 10.1093/nar/28.1.235 10592235 PMC102472

[B3] Billan S. Kaidar-Person O. Gil Z. (2020). Treatment after Progression in the Era of Immunotherapy. Lancet Oncol. 21 (10), e463. 10.1016/S1470-2045(20)30328-4 33002442

[B4] Cao Z. Zhang S. (2016). An Integrative and Comparative Study of Pan-Cancer Transcriptomes Reveals Distinct Cancer Common and Specific Signatures. Sci. Rep. 6, 33398. 10.1038/srep33398 27633916 PMC5025752

[B5] Cava C. Bertoli G. Colaprico A. Olsen C. Bontempi G. Castiglioni I. (2018). Integration of Multiple Networks and Pathways Identifies Cancer Driver Genes in Pan-Cancer Analysis. BMC Genomics 19 (1), 25. 10.1186/s12864-017-4423-x 29304754 PMC5756345

[B6] Chan T. A. Yarchoan M. Jaffee E. Swanton C. Quezada S. A. Stenzinger A. (2019). Development of Tumor Mutation burden as an Immunotherapy Biomarker: Utility for the Oncology Clinic. Ann. Oncol. 30 (1), 44–56. 10.1093/annonc/mdy495 30395155 PMC6336005

[B7] Clarke A. J. Simon A. K. (2019). Autophagy in the Renewal, Differentiation and Homeostasis of Immune Cells. Nat. Rev. Immunol. 19 (3), 170–183. 10.1038/s41577-018-0095-2 30531943

[B8] Consortium G. T. (2020). The GTEx Consortium Atlas of Genetic Regulatory Effects across Human Tissues. Science 369 (6509), 1318–1330. 10.1126/science.aaz1776 32913098 PMC7737656

[B9] Dalton D. K. Pitts-Meek S. Keshav S. Figari I. S. Bradley A. Stewart T. A. (1993). Multiple Defects of Immune Cell Function in Mice with Disrupted Interferon-Gamma Genes. Science 259 (5102), 1739–1742. 10.1126/science.8456300 8456300

[B10] Döhner H. Miguel A. S. Sanz A. Deeren D. Demeter J. Anagnostopoulos A. (2016). Phase Iii Randomized Trial of Volasertib Plus Low-Dose Cytarabine (Ldac) versus Placebo Plus Ldac in Patients Aged?65 Years with Previously Untreated Aml, Ineligible for Intensive Therapy. Available from https://library.ehaweb.org/eha/2016/21st/135257/hartmut.dhner.phase.iii.randomized.trial.of.volasertib.plus.low-dose.html?f=m3.

[B11] Finn O. J. (2008). Cancer Immunology. N. Engl. J. Med. 358 (25), 2704–2715. 10.1056/NEJMra072739 18565863

[B12] Forli S. (2015). Charting a Path to Success in Virtual Screening. Molecules 20 (10), 18732–18758. 10.3390/molecules201018732 26501243 PMC4630810

[B13] Goldman M. J. Craft B. Hastie M. Repečka K. McDade F. Kamath A. (2020). Visualizing and Interpreting Cancer Genomics Data via the Xena Platform. Nat. Biotechnol. 38 (6), 675–678. 10.1038/s41587-020-0546-8 32444850 PMC7386072

[B14] Guo J. C. Wei Q. S. Dong L. Fang S. S. Li F. Zhao Y. (2021). Prognostic Value of an Autophagy-Related Five-Gene Signature for Lower-Grade Glioma Patients. Front. Oncol. 11, 644443. 10.3389/fonc.2021.644443 33768004 PMC7985555

[B15] Hänzelmann S. Castelo R. Guinney J. (2013). GSVA: Gene Set Variation Analysis for Microarray and RNA-Seq Data. BMC Bioinformatics 14, 7. 10.1186/1471-2105-14-7 23323831 PMC3618321

[B16] Havel J. J. Chowell D. Chan T. A. (2019). The Evolving Landscape of Biomarkers for Checkpoint Inhibitor Immunotherapy. Nat. Rev. Cancer 19 (3), 133–150. 10.1038/s41568-019-0116-x 30755690 PMC6705396

[B17] Inc., T.C. FDA-Approved & Pharmacopeia Drug Library (2021). Available from https://www.targetmol.com/compound-library/FDA-Approved%20&%20Pharmacopeia%20Drug%20Library#.

[B18] Kawabata T. (2010). Detection of Multiscale Pockets on Protein Surfaces Using Mathematical Morphology. Proteins 78 (5), 1195–1211. 10.1002/prot.22639 19938154

[B19] Lavecchia A. Di Giovanni C. (2013). Virtual Screening Strategies in Drug Discovery: a Critical Review. Curr. Med. Chem. 20 (23), 2839–2860. 10.2174/09298673113209990001 23651302

[B20] Le Guerroué F. Eck F. Jung J. Starzetz T. Mittelbronn M. Kaulich M. (2017). Autophagosomal Content Profiling Reveals an LC3C-dependent Piecemeal Mitophagy Pathway. Mol. Cel 68 (4), 786–e6. 10.1016/j.molcel.2017.10.029 29149599

[B21] Lemery S. Keegan P. Pazdur R. (2017). First FDA Approval Agnostic of Cancer Site - when a Biomarker Defines the Indication. N. Engl. J. Med. 377 (15), 1409–1412. 10.1056/NEJMp1709968 29020592

[B22] Li K. Luo H. Huang L. Luo H. Zhu X. (2020). Microsatellite Instability: a Review of what the Oncologist Should Know. Cancer Cel Int 20, 16. 10.1186/s12935-019-1091-8 PMC6958913 31956294

[B23] Martincorena I. Campbell P. J. (2015). Somatic Mutation in Cancer and normal Cells. Science 349 (6255), 1483–1489. 10.1126/science.aab4082 26404825

[B24] Merck P. (2020). (package Insert). Available from https://www.merck.com/product/usa/pi_circulars/k/keytruda/keytruda_pi.pdf.

[B25] Mo Z. Li P. Cao Z. Zhang S. (2021). A Comprehensive Pan-Cancer Analysis of 33 Human Cancers Reveals the Immunotherapeutic Value of Aryl Hydrocarbon Receptor. Front. Immunol. 12, 564948. 10.3389/fimmu.2021.564948 34290693 PMC8287657

[B26] Morishita H. Mizushima N. (2019). Diverse Cellular Roles of Autophagy. Annu. Rev. Cel Dev Biol 35, 453–475. 10.1146/annurev-cellbio-100818-125300 31283377

[B27] Newman A. M. Steen C. B. Liu C. L. Gentles A. J. Chaudhuri A. A. Scherer F. (2019). Determining Cell Type Abundance and Expression from Bulk Tissues with Digital Cytometry. Nat. Biotechnol. 37 (7), 773–782. 10.1038/s41587-019-0114-2 31061481 PMC6610714

[B28] Norman P. J. Norberg S. J. Guethlein L. A. Nemat-Gorgani N. Royce T. Wroblewski E. E. (2017). Sequences of 95 Human MHC Haplotypes Reveal Extreme Coding Variation in Genes Other Than Highly Polymorphic HLA Class I and II. Genome Res. 27 (5), 813–823. 10.1101/gr.213538.116 28360230 PMC5411776

[B29] Nusinow D. P. Szpyt J. Ghandi M. Rose C. M. McDonald E. R. Kalocsay M. (2020). Quantitative Proteomics of the Cancer Cell Line Encyclopedia. Cell 180 (2), 387–e16. 10.1016/j.cell.2019.12.023 31978347 PMC7339254

[B30] Ohsumi Y. (2012). Yoshinori Ohsumi: Autophagy from Beginning to End. Interview by Caitlin Sedwick. J. Cel Biol 197 (2), 164–165. 10.1083/jcb.1972pi PMC3328387 22508506

[B31] Reinhold W. C. Sunshine M. Liu H. Varma S. Kohn K. W. Morris J. (2012). CellMiner: a Web-Based Suite of Genomic and Pharmacologic Tools to Explore Transcript and Drug Patterns in the NCI-60 Cell Line Set. Cancer Res. 72 (14), 3499–3511. 10.1158/0008-5472.CAN-12-1370 22802077 PMC3399763

[B32] Ritchie M. E. Phipson B. Wu D. Hu Y. Law C. W. Shi W. (2015). Limma powers Differential Expression Analyses for RNA-Sequencing and Microarray Studies. Nucleic Acids Res. 43 (7), e47. 10.1093/nar/gkv007 25605792 PMC4402510

[B33] Ru B. Wong C. N. Tong Y. Zhong J. Y. Zhong S. S. W. Wu W. C. (2019). TISIDB: an Integrated Repository portal for Tumor-Immune System Interactions. Bioinformatics 35 (20), 4200–4202. 10.1093/bioinformatics/btz210 30903160

[B34] Sanz-Garcia E. Argiles G. Elez E. Tabernero J. (2017). BRAF Mutant Colorectal Cancer: Prognosis, Treatment, and New Perspectives. Ann. Oncol. 28 (11), 2648–2657. 10.1093/annonc/mdx401 29045527

[B35] Shears S. B. (2001). Assessing the Omnipotence of Inositol Hexakisphosphate. Cell Signal 13 (3), 151–158. 10.1016/s0898-6568(01)00129-2 11282453

[B36] Slobodkin M. R. Elazar Z. (2013). The Atg8 Family: Multifunctional Ubiquitin-like Key Regulators of Autophagy. Essays Biochem. 55, 51–64. 10.1042/bse0550051 24070471

[B37] Squibb B.-M. (2020). Ipilimumab (Package Insert). Available from https://packageinserts.bms.com/pi/pi_yervoy.pdf.

[B38] Squibb B.-M. (2020). Nivolumab (Package Insert). Available from https://packageinserts.bms.com/pi/pi_opdivo.pdf.

[B39] Steed M. E. Rybak M. J. (2010). Ceftaroline: a New Cephalosporin with Activity against Resistant Gram-Positive Pathogens. Pharmacotherapy 30 (4), 375–389. 10.1592/phco.30.4.375 20334458

[B40] Tooze S. A. Yoshimori T. (2010). The Origin of the Autophagosomal Membrane. Nat. Cel Biol 12 (9), 831–835. 10.1038/ncb0910-831 20811355

[B41] Wallace A. C. Laskowski R. A. Thornton J. M. (1995). LIGPLOT: a Program to Generate Schematic Diagrams of Protein-Ligand Interactions. Protein Eng. 8 (2), 127–134. 10.1093/protein/8.2.127 7630882

[B42] Wang B. Niu D. Lai L. Ren E. C. (2013). p53 Increases MHC Class I Expression by Upregulating the Endoplasmic Reticulum Aminopeptidase ERAP1. Nat. Commun. 4, 2359. 10.1038/ncomms3359 23965983 PMC3759077

[B43] Wang Y. Lin K. Xu T. Wang L. Fu L. Zhang G. (2021). Development and Validation of Prognostic Model Based on the Analysis of Autophagy-Related Genes in colon Cancer. Aging (Albany NY) 13 (14), 19028–19047. 10.18632/aging.203352 34315829 PMC8351728

[B44] Xia H. Green D. R. Zou W. (2021). Autophagy in Tumour Immunity and Therapy. Nat. Rev. Cancer 21 (5), 281–297. 10.1038/s41568-021-00344-2 33758415 PMC8087647

[B45] Xu H. Chun T. Choi H. J. Wang B. Wang C. R. (2006). Impaired Response to Listeria in H2-M3-Deficient Mice Reveals a Nonredundant Role of MHC Class Ib-specific T Cells in Host Defense. J. Exp. Med. 203 (2), 449–459. 10.1084/jem.20051866 16476767 PMC2118219

[B46] Xu Z. Wu Z. Zhang J. Zhou R. Ye L. Yang P. (2020). Development and Validation of an Oxidative Phosphorylation-Related Gene Signature in Lung Adenocarcinoma. Epigenomics 12 (15), 1333–1348. 10.2217/epi-2020-0217 32787683

[B47] Yamamoto H. Imai K. (2019). An Updated Review of Microsatellite Instability in the Era of Next-Generation Sequencing and Precision Medicine. Semin. Oncol. 46 (3), 261–270. 10.1053/j.seminoncol.2019.08.003 31537299

[B48] Yang A. Herter-Sprie G. Zhang H. Lin E. Y. Biancur D. Wang X. (2018). Autophagy Sustains Pancreatic Cancer Growth through Both Cell-Autonomous and Nonautonomous Mechanisms. Cancer Discov. 8 (3), 276–287. 10.1158/2159-8290.CD-17-0952 29317452 PMC5835190

[B49] Yoshihara K. Shahmoradgoli M. Martínez E. Vegesna R. Kim H. Torres-Garcia W. (2013). Inferring Tumour Purity and Stromal and Immune Cell Admixture from Expression Data. Nat. Commun. 4, 2612. 10.1038/ncomms3612 24113773 PMC3826632

[B50] Zou W. (2005). Immunosuppressive Networks in the Tumour Environment and Their Therapeutic Relevance. Nat. Rev. Cancer 5 (4), 263–274. 10.1038/nrc1586 15776005

[B51] Zou W. Wolchok J. D. Chen L. (2016). PD-L1 (B7-H1) and PD-1 Pathway Blockade for Cancer Therapy: Mechanisms, Response Biomarkers, and Combinations. Sci. Transl Med. 8 (328), 328rv4. 10.1126/scitranslmed.aad7118 PMC4859220 26936508

